# ‘At this [*adherence*] club, we are a family now’: A realist theory-testing case study of the antiretroviral treatment adherence club, South Africa

**DOI:** 10.4102/sajhivmed.v20i1.922

**Published:** 2019-06-26

**Authors:** Ferdinand C. Mukumbang, Brian van Wyk, Sara Van Belle, Bruno Marchal

**Affiliations:** 1 School of Public Health, University of the Western Cape, Cape Town, South Africa; 2 Department of Public Health, Institute of Tropical Medicine, Antwerp, Belgium

**Keywords:** Antiretroviral Treatment, Adherence Club, Medication Adherence, Retention in Care, Realist Evaluation, South Africa

## Abstract

**Background:**

An estimated 7.9 million people were living with HIV in South Africa in 2017, with 63.3% of them remaining in antiretroviral therapy (ART) care and 62.9% accessing ART. Poor retention in care and suboptimal adherence to ART undermine the successful efforts of initiating people living with HIV on ART. To address these challenges, the antiretroviral adherence club intervention was designed to streamline ART services to ‘stable’ patients. Nevertheless, it is poorly understood exactly how and why and under what health system conditions the adherence club intervention works.

**Objectives:**

The aim of this study was to test a theory on how and why the adherence club intervention works and in what health system context(s) in a primary healthcare facility in the Western Cape Province.

**Method:**

Within the realist evaluation framework, we applied a confirmatory theory-testing case study approach. Kaplan–Meier descriptions were used to estimate the rates of dropout from the adherence club intervention and virological failure as the principal outcomes of the adherence club intervention. Qualitative interviews and non-participant observations were used to explore the context and identify the mechanisms that perpetuate the observed outcomes or behaviours of the actors. Following the retroduction logic of making inferences, we configured information obtained from quantitative and qualitative approaches using the intervention–context–actor–mechanism–outcome heuristic tool to formulate generative theories.

**Results:**

We confirmed that patients on ART in adherence clubs will continue to adhere to their medication and remain in care because their self-efficacy is improved; they are motivated or are being nudged.

**Conclusion:**

A theory-based understanding provides valuable lessons towards the adaptive implementation of the adherence club intervention.

## Background

South Africa has the largest AIDS epidemic in the world, with an estimated 7.9 million people living with HIV (PLHIV) as of 2017.^1^ The South African health system currently runs the largest antiretroviral therapy (ART) programme in the world.^2^ Although major successes have been achieved by the South African health system in responding to the HIV epidemic, yet challenges remain. These challenges are reflected in the sustained high HIV incidence rates,^3^ poor retention in ART care and suboptimal adherence to medication.^2^ Poor retention in care and suboptimal adherence to medication threaten the success of the South African national ART programme. With an estimated 4.44 million people initiated on ART to date^1^ and the recent adoption of the ‘test and treat’ approach,^4^ the need for sustainable programmes to improve the retention in care and adherence to ART is critical.

A consolidated version of the World Health Organization’s (WHO’s) 2015 HIV treatment guideline recommends the use of differentiated care models to improve the access and quality of treatment and care services for PLHIV.^4^ Differentiated models adapt HIV treatment services to specific patient populations and contexts, rather than adopting a one-size-fits-all approach.^5^ By tailoring services according to the needs of different patient groups, reducing clinic contact and relying on community-based services for quick medication access for the treatment of mature patients, these models increase the capacity and efficiency of ART services.

The adherence club intervention is an example of a differentiated care model designed to streamline ART care for adults (18+ years), treatment-experienced patients on first-line treatment with a good clinic attendance record and evidence of medication adherence.^6,7^ Through quick group consultations, convenient medication pickup processes and direct access to a clinician when needed, the adherence club drastically reduces the waiting times of the patients. The adherence club intervention also provides a conducive social environment to encourage patient interactions with peers. The adherence club intervention has been described in greater detail elsewhere.^8^

The evidence supporting the effectiveness of the adherence club intervention^9,10,11,12^ and its cost-effectiveness^13^ has prompted plans to roll out the intervention nationwide.^2^ Nevertheless, there is a limited theory-based understanding of *how* and *why* the adherence club intervention works and in what health system context(s). To this end, a realist evaluation of the adherence club intervention was commissioned.^14^ In this article, we report on the process of testing the hypothesis (initial programme theory) of how and why the adherence club intervention is expected to work under real-life implementation conditions.

## Methodological approach

The realist evaluation, a theory-driven approach, guided the inquiry.^15^ The goal of realist evaluation is about learning ‘for whom, in what circumstances, and in what respects a programme works’,^15,16^ through identifying, testing and refining programme theories. Therefore, realist evaluation starts with an initial programme theory and the goal is to obtain a more refined programme theory.

Programmes work with the acquiescence of participants (actors),^17^ and provide resources, opportunities or constraints of some kind that influence the target person’s decision-making.^18^ Therefore, understanding why a programme works (or not) rests on the ability of the evaluator to explain the decision-making process of the relevant actors regarding the resources, opportunities and constraints that the programme provides to the relevant actors.

**FIGURE 1:**
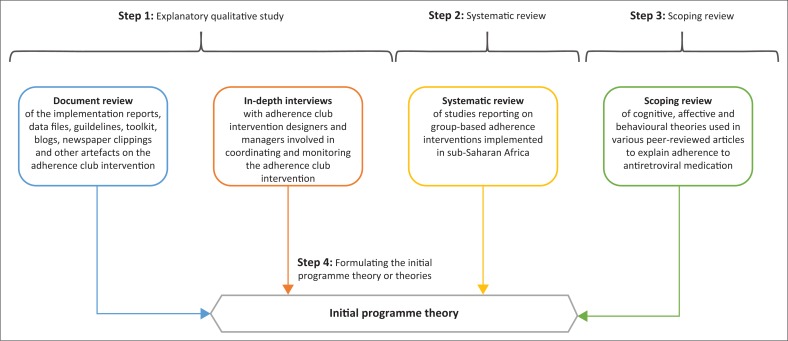
Sources of information towards formulating the initial programme theory.

Identifying the important generative mechanisms (social and psychological drivers) of a programme is not enough to explain how and why a programme works (or not).^19^ For an intervention to work, it must influence the reasoning (mechanism) of the targeted actors to cause them to adopt an intended behaviour that, in a specific context, will lead to a specific outcome. Therefore, realists assume that an outcome (O) is generated by a mechanism (M) being triggered in context (C) through an actor (A) when an intervention (I) is implemented. This captures how, why, for whom and in what circumstances programmes work. Formulating realist theories is, therefore, achieved through the formation of intervention–context–actor–mechanism–outcome (ICAMO) configurations.^20,21^ The ICAMO configuration is a modification of context–mechanism–outcome (CMO), originally proposed by Pawson and Tilley^22^ as the heuristic tool for the development of realist theories. In the first phase of this project, we elicited an initial programme theory of the adherence club intervention with information obtained from four sources^23,24,25^ (see Figure 1).

We applied the ICAMO heuristic tool to configure the programme theory by applying the logic of retroduction – mechanism-centred analysis and conceptualisation – to make inferences. Testable hypotheses were distilled from the configurational map,^8^ an approach to causality whereby outcomes are considered to follow from the alignment of a specific combination of attributes,^16^ using the ‘if … then … because’ phrase (Box 1).

BOX 1 Initial programme theory of the adherence club intervention represented by two tentative theories (hypotheses).

**Initial Programme Theory 1**

*If* adult (18+ years), clinically ‘stable’ patients with evidence of good clinic attendance are group-managed, receive quick symptom checks, quick access to medication, consistent counselling and social support from the peer counsellor,

*Then* they are likely to adhere to medication and remain in care,

*Because* they develop a group identity, which improves their perceived social support and increases satisfaction and trust, and acquire knowledge, which helps them to understand their perceived threat and perceived benefits and improves their self-efficacy. As a result, they become encouraged, empowered and motivated, thus more likely to remain in care and adhere to the treatment.

**Initial Programme Theory 2**

*If* operational staff receive goals and targets set to continuously enrol patients in the adherence club and monitor their participation through strict standard operating practices (the promise of exclusion in the event of missed appointment and active patient tracing),

*Then* patients are likely to adhere to medication and remain in care,

*Because* they fear losing the benefits (easy access to medication, peer support, reduced waiting times, and 2-month ART collection) of the club system and are coerced through adhesive club rules. As a result, they are nudged to remain in care and adhere to the treatment, which might decongest the health facility.

The aim of this study was to test these initial programme theories in a primary healthcare facility running the adherence club programme, with the goal of obtaining a more refined programme theory of the adherence club intervention. In conducting and reporting the findings of the study, we followed the RAMESES II reporting standards for realist evaluation developed by Wong et al.^26^

## Research design

This study is framed within the realist evaluation approach. We sought to test the initial programme theory of the adherence club intervention in a real-life implementation situation to verify, refute and/or modify the initial programme theory of the adherence club intervention. To this end, we adopted an explanatory theory-building case approach and the multiple embedded case study design.^27^ Facility Y was considered the case and the unit of analysis, with each of its ART clubs being sub-units embedded in the case.

According to Creswell and Plano Clark,^28^ cases selected for case study research could be identified as typical, deviant or crucial. Facility Y was selected as a deviant case, a most likely case shown to be negative with regard to the phenomenon under consideration. To this end, we considered Facility Y for testing the initial programme theory of the adherence club intervention. This facility has retention in care rates of only 63.0% based on the routine monitoring and evaluation data on the adherence club intervention of 2015. Although Facility Y was selected for the first phase rollout of the adherence club intervention in 2012 along with other clinics in the health sub-districts, the intervention only rolled out in September 2014. Reasons were challenges related to lack of physical space and poor buy-in from the facility healthcare providers.

## Study setting

Facility Y is a provincial primary healthcare facility providing primary healthcare to the surrounding communities. Staff provide first-level and some second-level care, including a 24-h emergency service. Housed in a separate building from the main clinic, is an accredited ART initiation and on-going management site, which operates Mondays to Fridays providing treatment and care services to PLHIV and those infected with tuberculosis (TB). Patients who are co-infected with HIV and TB can easily access both services, as those with HIV and TB share the same waiting area and are seen by the same counsellors.

Because of a lack of proper structures such as physical meeting space, the programme could not be implemented at the scheduled time. Following the construction of a makeshift building for club activities, the intervention was initiated at the facility. While conducting a preliminary qualitative exploration for the suitability of Facility Y for our study, we uncovered that the adherence club programme was poorly implemented because of poor buy-in from the staff members, who failed to identify how the intervention would benefit them and/or improve the overall delivery of ART services to the patients. They perceived the adherence club intervention as extra work in their already busy schedule. Consequently, even when a makeshift building was constructed for the adherence club activities, the programme struggled to function properly.

When the sub-district managers identified the problem through routine monitoring and reporting, a nurse was identified and trained in the implementation and execution of the adherence club programme to champion the intervention at Facility Y. This nurse subsequently ran workshops and meetings with the other ART care providers at the facility expounding on the advantages of the intervention to the patients, the healthcare workers themselves and the clinic. This strategy led to an overall improvement of the level of buy-in, uptake and implementation of the adherence club intervention. To date, an estimated 50 clubs with 25–35 patients each have been established at the facility.

## Research methods

We combined a retrospective cohort analysis and an explanatory qualitative approach to data collection. Using a sequential explanatory approach,^29^ we first collected the quantitative data, which informed the retention in care and suppressive adherence to medication outcomes at the facility. The quantitative data collection was followed by qualitative methods (non-participant observation, in-depth interviews and focus group discussions) aimed at informing the nature of the outcomes obtained. The combination of qualitative and quantitative methods allowed us to explore the important contextual elements that influence the implementation of that adherence club intervention, the mechanisms that the intervention introduces and the emergent outcome patterns. The multi-method approach also allowed for triangulation.

## Selection of respondents

Regarding the retrospective cohort arm of the study, our goal was to identify a typical ‘mature’ adherence club, that is, a club that reached its maximum capacity of 30–35 patients. Firstly, we selected all the clubs that had opened in 2014, and then identified the clubs that had reached their maximum capacity (35 patients per club) in the same year – seven clubs were identified. We purposefully decided to select two clubs to allow us to compare their retention in care and ability to enhance adherence to medication. These two clubs were randomly selected from the identified seven to conduct the survival analysis using the fishbowl or lottery method – without replacing.^30^ All the patients in the cohort of each of the selected adherence clubs (35 per club) were included in the cohort analysis.

Regarding the interview process, we included all the operational staff working on the adherence club programme at the facility, which comprised an adherence club nurse, who heads the adherence club programme, and three lay counsellors. We applied a purposive sampling approach to select six participants to be interviewed from the two adherence clubs sampled for the quantitative retrospective arm of the study. Our goal was to obtain at least one male and one female from each of the clubs. We also included two patients who were members of the adherence club, but had been asked to return to the standard care scheme at the main facility because they failed to follow all the club rules. Table 1 elaborates on the characteristics of the participants who were interviewed.

**TABLE 1:** Characteristics of study participants for the qualitative interviews.

Stakeholder	Number of participants	Time on adherence club
Nurses	1	Nurse 1 – 2012
Counsellors (club facilitators)	3	Counsellor 1 – 2010
		Counsellor 2 – 2010
		Counsellor 3 – 2014
Patients (club members)	4	Patient 1 (female) – 2014
		Patient 2 (male) – 2014
		Patient 3 (female) – 2014
		Patient 4 (male) – 2014
Patients (former club members)	2	Ex-member 1 (female) – 2014
		Ex-member 2 (male) – 2014

## Data collection process

We used the two sampled clubs as the focus of data collection for the observation. The quantitative data were extracted from the adherence club registers at the clinic. In the Western Cape, information relating to retention in care is registered using the modalities outlined in Table 2.

**TABLE 2:** Modalities defining adherence club attendance.

Recorded outcome	Outcome event
DNA	*Did not attend* club session or sent a buddy within 5 days after the club sessions
BTC	*Back to Clinic* – exiting the club for medical reasons and reinstated in routine standard of care
TFOC	*Transferred out to a different club* – patient is transferred out to another club in the same facility
TFO	*Transfer out* – patient is leaving the facility completely and will attend a clinic elsewhere
RIP	*Rest in peace* – patient has died

Concerning retention in care, the patients were considered not retained in care if they did not attend a club session or sent a ‘buddy’ and were sent back to the clinic. Patients were considered censored if they were transferred out to a different club or clinic, or died. For adherence to medication, when a patient was not attending that club for whatever reason, they were considered censored. The viral load of the patients is used as a proxy indicator of adherence to medication. Non-adherence was identified as any reading > 400 copies/cm^3^ and adherence was represented as LDL (lower than detectable reading).

We conducted four non-participant observations^31^ of the adherence club meetings, where we observed club sessions without interfering in any of the processes. These included two sessions of exclusive medication collection and a blood sample collection plus medication collection. The goal of the non-participant observation was to obtain insights into events and activities and the meanings that the club members attach to the sessions. We captured the dynamics of interactions of the group members with each other and with care providers in our field notes. During each observation session, we took detailed field notes.

After the non-participant observations, we conducted realist interviews – a theory-driven approach to interviewing^32,33^ – to uncover the causal relationship of aspects related to the implementation of the adherence club intervention. The investigation looked at the relevant context, generative mechanisms and emerging outcomes in relation to the patients (actors). Pawson^32^ advises that in applying the realist interviewing approach, *the researcher’s theory is the subject matter of the interview, and the subject is there to confirm or falsify and, above all, to refine that theory*. He also suggests that the care providers are versed in issues around the context and outcome of the intervention, while the patients, being the principal actors in the intervention, can provide mechanism-related attributes.^32^ The patient interviews were conducted after the second non-participant observation.

The quantitative data were captured using Microsoft Excel and prepared for analysis using the Statistical Package for Social Sciences (IBM SPSS) version 24. The field notes from the non-participant observations were also developed into transcripts. The audio-recorded interviews were transcribed verbatim by a professional transcriber and prepared for analysis. Atlas.ti version 7 was used to manage the field notes and interview transcripts.

## Data analysis

To identify and describe the outcome patterns of the adherence club intervention regarding retention in care and adherence to medication, we used the Kaplan–Meier method – the probability of surviving in a given time while considering time in many small intervals.^34^ This method was suitable because it allowed us to estimate the rate at which patients remained in care and the rate at which they maintained a viral load lower than detectable (< 400 copies/mm³ of blood) at 6-month intervals, covering a 24-month period.

The analysis of the qualitative data involved the coding of the realist interview (semi-structured) transcripts. The coding process was done by the first author who has extensive knowledge on the subject matter,^35^ with a previously validated coding frame by four authors that was based on the initial programme theory (Appendix 1). After the coding process, we classified the themes as a mechanism, context, actors, intervention and outcomes.

## Ethical consideration

Regarding the study participants, we first provided the participants with an information sheet for the project. This was followed by a verbal explanation of the role of the participants and the significance of their participation. The participants were required to sign an informed consent form. We promised and ensured confidentiality and anonymity by identifying the participants using pseudo names and password-protecting all the study related files. This study is part of a larger project ‘A realist evaluation of the antiretroviral treatment adherence club program in selected primary healthcare facilities in the metropolitan area of Western Cape Province, South Africa’, which has received ethical clearance from the Higher Degree’s Committee of the University of the Western Cape. In addition, we obtained ethical clearance from the Provincial Department of Health of the Western Cape Province. We also obtained permission from the facility heads.

## Results

The findings are presented in relation to the two initial programme theories.

### Qualitative findings

#### Context

Context relates to important conditions relevant to the implementation of the adherence club, which includes buy-in from health workers, clinic organisation, the number of clubs run by the facility, staffing dynamics, availability of resources (including human resources), pre-club preparations (including teamwork) done by the club team and individual patients’ attributes.

#### Buy-in from health workers

Although buy-in could be identified as an important mechanism for the implementation of the adherence club intervention, it also constitutes an important context element for its day-to-day functioning. Our analysis revealed that buy-in was not always obtained from all the operational managers when the programme was initially rolled out in the facility. One of the counsellors explained the situation below:

‘When the idea of clubs came in 2011, we did not like the idea because we knew that it would be more work for us. It meant that we had to do our normal patient counselling including the TB patients and then still organise the clubs. We were not happy about it … But when sister came, she explained that the clubs will reduce the waiting times of the patients because we were always complaining “we were working so slow, the time periods, waiting periods is long”.’ (Counsellor 2, female)

Our study participants revealed that at the time of the study, there was a good buy-in from the operational staff regarding the implementation of the adherence club programme. This buy-in prompts them to work beyond the call of duty. For instance, a counsellor explained that rather than start the adherence club sessions at 8 am, as originally scheduled, they start the club activities at 7 am to allow the patients to finish at the club and still make it to their workplaces on time. This is what the nurse had to say:

‘We have to have buy-in from everybody, so I also had to speak to the pharmacists, telling them, “This is the plan, this is the reason” and tell them how they are going to *benefit* by fewer patients waiting in their waiting area.’ (Nurse 1, female)

#### Integrated care

Integrated care means providing services relating to not only ART, but also services of other non-communicable chronic diseases, such as hypertension, diabetes and epilepsy. Patients having any other illness and who are on ART in the adherence club are also provided with services to manage the concomitant non-communicable chronic diseases. This context encourages the successful implementation of the adherence club regarding patients with comorbidities. The nurse participant explained how the notion of integrated care provides for a conducive environment for patients with other comorbidities along with living with HIV:

‘What we have also done is now all the patients, because we provide a holistic, integrated service in this department, we have made a chronic club [*patients with concomitant HIV and non-infectious chronic diseases*]. We have three chronic clubs. If you have hypertension or diabetes, then we will put you together in one club. So, we know when *those* patients come, we measure their blood pressures, we will test their sugar levels, and we will send them for their yearly eye testing. We also do their feet exam, so that they are also not disadvantaged.’ (Nurse 1, female)

#### Availability of conducive physical space

The availability of appropriate physical space where the adherence club sessions could be conducted is an important context condition. In fact, the lack of a physical structure was one of the main reasons why the adherence club programme at Facility Y only commenced in 2014 when a makeshift building was constructed. Some of the providers suggested that having a separate unit to run the adherence club programme is ideal. This was confirmed by the comments of the adherence club nurse who suggested that having a separate, dedicated space for ART adherence clubs provides an air of privacy for the patients:

‘We have a separate space at the back for club activities. So, they have got their own privacy and their own space. They have that freedom and it is not with everybody else.’ (Nurse 1, female)

#### Availability of a programme champion

A champion is someone who is dedicated to the success of a programme and closely monitors the implementation and execution of every aspect of the programme. Having a programme champion is identified, therefore, as an important context element for the successful execution of the adherence club intervention. It triggers the required mechanisms to ensure retention in care and adherence to medication. A participant said the following to capture the importance of having a programme champion, especially with regard to initiating and sustaining buy-in from other operational staff:

‘When I came here, I received training and I wanted to find out more about this club. I received training from an NGO and they told us … what the goal of the club is, what is the aim, and then once I got the buy-in and the training, then I realised the *benefits* of it. I then encouraged my staff and told them “The more patients we put in the club, the fewer patients we have to see”. Because the staff is always complaining, “There are many patients”. They are overworked, they are exhausted, there are staff shortages, their morale is low and it is just too much. So, I said okay, I had several meetings with them. I said, “Guys, we need to pull together because we are not going to get more staff. The only way to decongest the people waiting in our clinic is to put them in a club because if they are in the club, we will have fewer people waiting here, fewer people that you have to see”. So then, everybody was on board, everybody is excited and then we got a timetable. So, everybody was given a responsibility that we need to fill out the clubs, write out the scripts, fewer patients.’ (Nurse 1, female)

#### Teamwork

Working as a team is identified as a favourable condition for the successful implementation and execution of the adherence club programme. This is related to the fact that each member of the team has a role to play, and if any of the team members fail to deliver, then the execution of the intervention is affected, which also affects how the users take up the intervention. One of the counsellors explained how each of the operational staff executing their responsibilities as a team member ensures that the adherence club programme works seamlessly:

‘Teamwork is *very important* because if the Sister [*Nurse*] does not prescribe the medication, you cannot get the medication from Pharmacy, there are going to be delays or *no* medication given. Then the patients have to go sit in that mainstream, then what is the use of the club because then they have to go back to the waiting area.’ (Counsellor 1, female)

### Mechanisms

We identified possible mechanisms from the data, based on the two parts of the initial programme theory, and defined a ‘mechanism’ as a process of how subjects interpret and act upon the intervention.^19^

### Initial Programme Theory 1

Regarding Theory 1 (Box 1), we found the following elements in the interviews with the stakeholders.

#### Motivation

Motivation relates to the motive behind an individual’s involvement in an activity. In this case, the outcome of interest relates to ease of receiving ART and care services that fit into one’s lifestyle. This is especially applicable for newly diagnosed patients who are not attending adherence clubs as they are not yet eligible to attend, but are aware of the benefits that the adherence club offers. One of the counsellors indicated how patients are usually motivated to join the adherence club for easy access to treatment and care by meeting the adherence club criterion of achieving viral load suppression:

‘When we prepare them [*treatment-naïve patients*] for ARVs, we tell them “in six months if your viral load is suppressed then you are going be a VIP which means starting from today you are starting your medication, you need to take your medication *very well*. You are not going to come to the clinic monthly. You will come maybe four times but it will be less than five times a year to the clinic, we take blood only once a year.” You know so we are starting to buy them in when we are preparing them. This motivates them to achieve a low viral load.’ (Counsellor 3, female)

#### Perceived benefit

The perceived benefit relates to the awareness of the positive impact that the resources of the adherence club could have on fitting their ART into their lifestyles. These benefits were related to the prompt service delivery that they received through the adherence club. One of the patients expressed their perceived benefit of having quick access to see a clinician when they have any issue(s) requiring clinician consultations. This is expressed in the quote below:

‘If you are here and you want to see the doctor, one of the nurses goes inside and speaks to a doctor [*on your behalf*]. Then, they will just come and fetch you here and then you go straight to the doctor.’ (Patient 1, male)

#### Satisfaction

Satisfaction relates to the fulfilment of the patients’ desires, expectations and needs regarding the service delivery offered through the adherence club. One of the patients expressed their satisfaction with the adherence club by indicating that it is nice to have everything done within the club – a one-stop-shop. This is how some of the patients described the care they experienced in the club:

‘Here we are doing everything inside here, so it is nice. *It is very nice*. So, if someone wants to go back to work, she or he can go back to work. So, it is encouraging for the person to take their medication, it is encouraging for the person to come to the clinic.’ (Patient 1, male)

According to the providers, the patients’ experiences with the adherence club care leave them satisfied, which makes them potentially remain in care and adhere to their medication.

#### Bonding and group identifying formation

Bonding is the formation of a close relationship, especially through frequent or constant association. The literature says that the bonding process can be facilitated by shared common goals and characteristics.^36^ The bond formation between the club members is an important mechanism as it fosters sharing among the club patients. One of the patients explained how being in the club and meeting on a regular basis enhances a close association with each other that they are now a family. This is what a patient said about bonding:

‘Okay, you see here as we are at this club, we are a family now. We did not know each other, but now, as we are here, we know each other … This is my family. Some other people here, they do have families, but they do not tell their families that they have this.’ (Patient 2, female)

#### Perceived social support

Perceived social support speaks to the awareness of the positive impact that the moral, psychological or physical support has on the patients receiving care in the adherence club intervention. The nature of the support received in the adherence club is predominantly peer support. According to Lee and Lok,^36^ peers can provide companionship, stimulation, physical support, ego support and intimacy. One of the patients indicated the nature of support that he or she receives when they are in the adherence club re moral support through chatting with other club members:

‘When I am here [*in the club*], I feel de-stressed (relaxed) like I am staying at home. I think the club is the right thing, because we share some talks, “how is the medication you take?” I prefer the club than to talk with friends.’ (Patient 1, male)

#### Knowledge acquisition

Knowledge acquisition is an important cognitive mechanism related to the health talks that are provided by the club facilitators during each club meeting session. We found that the acquisition of knowledge by the patients improves their self-efficacy – the perception of their ability to perform activities related to the self-management of their disease.^37^ In the quote below, a patient explained some of the things that they learn during the adherence club sessions, some related to enhancing medication adherence and others with regard to preventing the spread of HIV:

‘In the club, they are open; they always tell us how to take the tablets, what you are supposed to do. Like now, they were telling us that if you have a partner, you must not sleep without a condom. You must always “condomise”. They were telling us about TB [*Tuberculosis*], the side effects of drinking alcohol whilst you are on medication and the side effects of smoking cigarettes.’ (Patient 3, female)

One of the respondents suggested how this education received by the patients translates into understanding:

‘When they receive the health talks, at least they will have a clear understanding of what to do and how the club will be benefitting them.’ (Counsellor 3, female)

Based on our observation of the club sessions, we noticed that the club facilitators also spent some time to remind the patients during the health talk of the rules and regulations guiding the adherence club. They emphasised the behaviours that could potentially lead to the patient being ousted from the club, indicating that the rules were being reinforced. One of the counsellors confirmed this observation:

‘Yes, the health talks have a very big impact, because it reminds them [*patients*] of the dos and don’ts because if we do not give the talks, patients will forget the rules.’ (Counsellor 1, female)

#### Trust

One of the counsellors explained the unspoken code of conduct or the psychological contract, which exists among the members of the adherence club and the counsellors. That is, club members and counsellors are not meant to disclose the status of patients outside the adherence club meeting or with a non-club member. One of the counsellors explained how the underlying code of conduct to maintain confidentiality in the adherence clubs promotes trust among the members:

‘They know that whatever happens here [*in the club*], it remains here. We do not go and share things that are discussed here in the community. For example, if one of the club members is a friend or a neighbour or whoever, maybe they go to church together. They know that they are not allowed to go and talk “that we are in the same club”. So, they know that one of the rules is like confidentiality. Whatever happens here, remains here.’ (Counsellor 2, female)

#### Perceived barriers

Perceived barriers to leave the club are related to the circumstances that cause patients to be aware of the advantages offered by the adherence club intervention in light of the challenges they face in regular clinic care. In the excerpt that follows, a lay counsellor explained how perceived barriers related to seeking permission from work to pick up their monthly ART from the healthcare facility. Patients in ART care, therefore, cherish the two monthly medication pickup and the quick services offered at the club (nullifying the need for asking for permission):

‘When you are in the care of the facility, you only get maybe one month [*medication supply*]. So, every time you have to ask for a day off, leave, or something for you to get your medication. So [*in the club*], it is much easier. It saves time and then you do not have to sit in the clinic for the *whole day*.’ (Counsellor 1, female)

The following excerpts outline aspects of the regular ART clinic that patients perceive as barriers to their ART care. Because the adherence club is meant to remove these barriers, it encourages them to remain in ART care under the clubs:

‘And waiting at the aisles is not so nice like here [*in the clubs*], because then you have to wait for the doctors and you have to wait for everything, your tablets, even to go to the Pharmacy, but here [*in the clubs*], you can just come and get your medication and weight and that is it.’ (Patient 4, male)

A patient also identified the potential of being stigmatised, which is part of the environment of the regular ART clinics as a perceived barrier. This barrier is minimal or absent in the adherence club:

‘Therefore, I have to stay stable, because we do not like to stand there in front [*in the regular clinic*] so that the people are going to judge us. So, I have to take my medication so that the people do not see me there [*in the regular clinic*]. I must stay here at the club.’ (Patient 5, female)

### Initial programme theory 2

#### Perceived coercion

Perceived coercion is the awareness of being compelled or pressured to do something. Although the club intervention is beneficial to the patients, there are also some managerial benefits, such as decongesting the facility. Thus, the club rules are there to ensure the smooth functioning of the intervention as well as to promote the success of the programme. Nevertheless, some patients might interpret some of the club rules as being coercive:

‘I know the club [*has*] got rules and like the one rule. It [*club rules*] helps a lot because then you must take your medication. It is necessary that you take it [*medication*]; otherwise, you will go back to where you came [*regular ART clinic*].’ (Patient 4, male)

Our observation revealed that the patients were being reminded of the club rules at every club visit. They are particularly reminded of the circumstances that could lead to being sent back to the main clinic care.

#### Fear

Fear relates to the awareness of the dangers of being returned to the mainstream care and experiencing the barriers that the adherence club intervention addressed. One of the patients explained how the fear of being sent back to the main ART scheme, characterised by long waiting times and frequent travels to the clinic, causes them to not afford to miss a club session:

‘So, you do not want to be sent back, because you know you will return to waiting for two hours for tablets. You have to wait there and you go to the Pharmacy, there will be a queue. You know that if I miss the appointment with the club, then they might send me back.’ (Patient 3, female)

#### Nudging

Nudging is the notion of being guided towards making decisions that are considered beneficial (to the patient), usually by the healthcare providers by presenting options in a specific way. By providing restricted options to the patients receiving care in the adherence club programme, they are nudged to acting in a particular way as guided by the resources and principles that are on offer at the adherence club. A patient suggested they are being *made* to attend their adherence club sessions and to take their medication through the rules of the club, because if the rules are not abided to, then they are sent back to the main clinic care:

‘They make you come to the clinic, and they make you take your medication, because if you are going back to the main clinic, my dear, you will stay there for the whole day in the main clinic. You come at half past six, you stand in a queue there at the reception, then at eight o’clock, they start giving your folders and then from there you go to the scale.’ (Patient 1, male)

### Outcome

The outcomes that are identified here are based on the findings of quantitative analysis (retrospective cohort analysis – primary outcomes) and emergent outcomes from the interview process that are challenging or complex to measure (these are not part of the primary outcomes of retention in care and adherence to medication). These outcomes include decongesting the healthcare facility and reducing the workload of the healthcare workers.

#### Decongestion of the facility

One of the emerging outcomes of the adherence club intervention is that it contributes to decongesting the healthcare facility. One of the participants explained how this is being achieved:

‘It [*the club*] is decongesting because remember, there are 35 patients per club. There are some days that we have two clubs. So, remember, if it is one day, every day 35 patients from the normal waiting area are being removed. So, they receive their medication and their treatment thus decongesting the waiting area … On days that there are two clubs per day, that is 70 patients out of your waiting area.’ (Nurse 1, female)

### Quantitative findings

The role of quantitative (extensive) methods in realist research is considered to be predominantly descriptive. To this end, our quantitative findings are mostly descriptive. Table 3 illustrates the characteristics of the participants of the two selected adherence clubs.

**TABLE 3:** Characteristics of patients in clubs A and B.

Total number	Club A	Club B
	35	37
**Gender**		
Males	9	12
Females	26	25
**Mean age**	32 (interquartile range 20–57)	30 (interquartile range 22–60)
**Marital status**		
Single	12	16
Married	13	10
Divorced	10	11
**Employment status**		
Unemployed	13	17
Employed	22	20

#### Retention in care

The combined retention in care within a 24-month period is 77.8%, with ‘club A’ registering a much lower retention in care rate (71.4%) compared to ‘club B’ (83.8%) (Table 4).

**TABLE 4:** Retention in care distributions in two adherence clubs at Facility Y.

Adherence club	Total number	Number of patients not retained in care	Patients retained	
			Number	%
Club A	35	10	25	71.4
Club B	37	6	31	83.8
Overall	72	16	56	77.8

**FIGURE 2:**
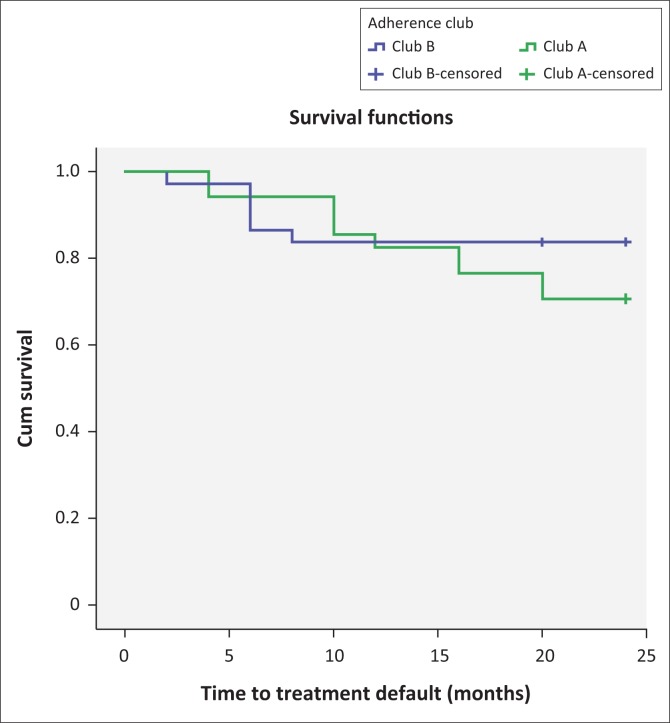
Survival distribution of patient retention in care in two adherence clubs at Facility Y.

The survival distributions of the patients receiving care in the two adherence clubs are shown in Figure 2. At 6 months, the retention in care rate of club A was 91.4% (95% CI, 75.8–97.8). At 12 months, the retention in care rate dropped to 77.1% (95% CI, 59.4–89). At 24 months, the rate decreased further to 65.7% (95% CI, 47.7–80.3). Club B registered better retention in care rates at 6, 12 and 24 months with values of 86.5% (95% CI, 70.4–94.9), 83.8% (95% CI, 67.3–93.2) and 81.1% (95% CI, 64.3–91.4), respectively. Fox et al. estimated a 6-year national retention in ART care in South Africa at 63.3%.^38^ Comparing this value to the overall retention in care value of Facility Y (77.8%), we suggested the 2-year retention in care rate of the facility is good.

We further conducted a log-rank test (Mantel–Cox) to determine whether the survival distributions of the two adherence clubs were statistically significantly different. A *p*-value of 0.255 showed that the survival distributions of the two adherence clubs were not statistically different, suggesting some level of constancy regarding retaining patients in care within the 24-month period. Nevertheless, club B showed signs of having stabilised, while a projection of club A showed that it had the potential of continuously losing patients.

#### Adherence to medication

Patients in club B showed better adherence to medication behaviours (89.2%) compared to those in club A (77.1%) (Table 5). The population-level adherence to medication based on the two sampled clubs was 83.3%.

**TABLE 5:** Adherence to medication distribution rates in two adherence clubs at Facility Y.

Adherence club	Total number	Number of patients non-adherent to ART	Adhering patients	
			Number	%
Club A	35	8	27	77.1
Club B	37	4	33	89.2
Overall	72	12	60	83.3

**FIGURE 3:**
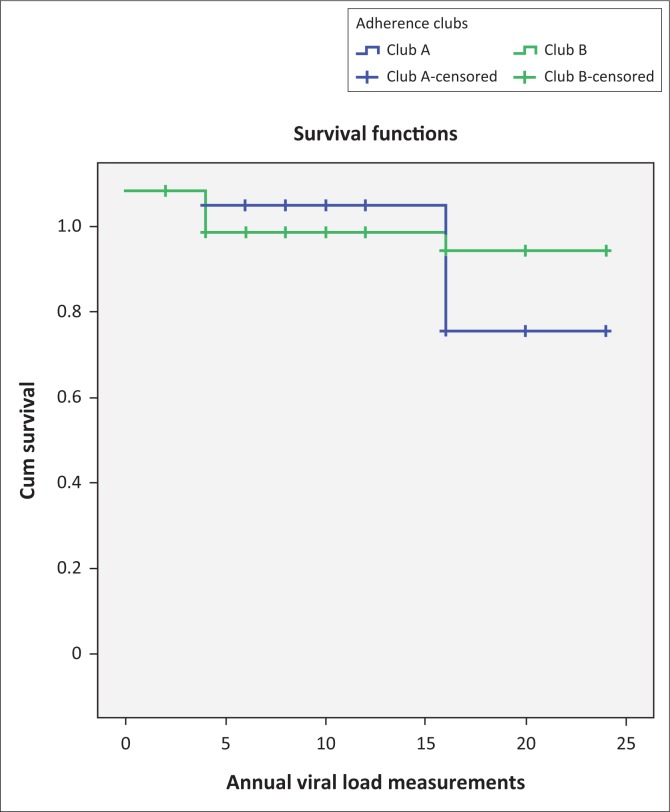
The survival distributions of the adherence behaviours of patients in the two adherence clubs at Facility Y.

Based on Figure 3, the adherence to medication of the club A members at 12 and 24 months was 97.1% (95% CI, 83.3–99.9) and 72.0% (95% CI, 54.0–85.2), respectively. At 12 months, club B members showed a slightly lower adherence rate of 91.7% (95% CI, 76.7–97.8) compared to club A members, but at 24 months, club B members showed a better retention in care rate of 88.4% (95% CI, 72.7–96.0) compared to club A members. According to the Human Sciences Research Council report of 2018, only 62.3% of all PLHIV in South Africa were virally suppressed.^1^ In comparison to the national ART adherence rate, we considered the 83.3% obtained as a ‘good’ adherence rate.

The log-rank test (Mantel–Cox) showed a value of 0.252, which indicates an overall consistency between the two clubs in enhancing adherence to medication among the patients using the intervention.

### Data synthesis

The data synthesis involved merging the data from the descriptive quantitative arm to the findings obtained from the thematic analysis of the qualitative transcripts in an attempt to refute, confirm or modify the initial programme theories. We formulated the ICAMO matrix following the different modalities of the adherence club intervention. This matrix is outlined in Table 6.

**TABLE 6:** Intervention–context–actor–mechanism–outcome matrix formulated from the study findings.

Intervention modalities	Context	Actor	Mechanism	Outcome
Club rules and regulations	- Standard operating protocol - Being reminded of the rules and regulations of the club - HIV policy	- Patient	- Perceived barriers - Perceived coercion - Perceived fear - Reinforcement - Nudged	- Nudged to adhere to club appointments
Group dynamics	- Availability of space for meeting - Relationship with other club members	- Patient - Group	- Perceived social support - Bonding and formation of group identity	- Better adherence resulting from developed self-efficacy
Health talks or education	- Good availability of personnel - Effective teamwork	- Patient	- Knowledge acquisition - Reinforcement of club rules and regulations	- Improved self-efficacy
Quick medication access	- Availability of medication - Eligibility criteria - The organisation of the pickup process and club sessions - Buy-in from care providers	- Patient	- Perceived benefit - Motivation - Satisfaction	- Adherence to medication related to medication availability
Prompt continuity of care	- Availability of clinicians - Staffing dynamics - The organisation of club activities - Buy-in from care providers	- Clinicians - Patient	- Trust - Satisfaction	- Retained in care through problem resolution
Club facilitator–patient relationship	- Staffing dynamics - Teamwork or collaboration - Buy-in from care providers	- Facilitator - Patient	- Trust - Perceived support	- Adherence to medication - Motivation - Retention in care
Overall intervention	- Availability of programme champion - Buy-in from care providers - Preparation and organisation	- Patients - Club teams	- Motivation - Self-efficacy - Satisfaction	- Improved retention in care and adherence to medication

In constructing the ICAMO matrix, we applied the configurational mapping approach, in which outcomes are considered to follow from the alignment of various interactive components. The retroduction logic informed this process. Retroduction is a form of inference that seeks to identify and verify mechanisms that are theorised to have generated the phenomena under study.^39^ Firstly, we paid attention to the outcomes of interest and then identified the mechanism(s) most associated with each outcome. This transfactual thinking approach^40^ helped us to identify mechanisms that were associated with the different modalities of the intervention and how these mechanisms relate to the different actors (patients, health professionals). Then, we examined the context in which the mechanisms are contingent to perpetuate the observed outcome as informed by the data. Thirdly, we confirmed each ICAMO chain by applying counterfactual thinking (creating possible alternatives) to trace the various pathways (demi-regularities).^40,41^

**FIGURE 4:**
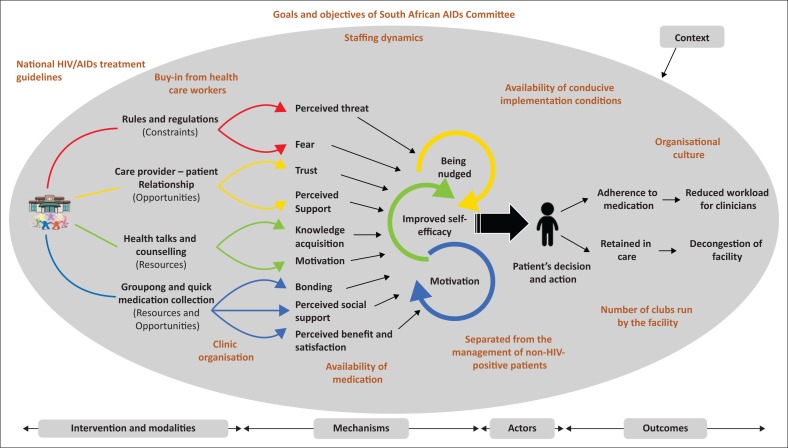
Modified programme theory.

While having ICAMO links, such as in Table 6, is useful, we constructed a configurational map to obtain a bigger picture. According to Byng et al.^42^ the bigger picture adds value to understanding the programme theory. The result of this exercise is a model illustrating how adherence clubs contribute to adherence and retention in care (Figure 4).

Our analysis reveals that the two theories identified as initial programme theories complement each other to provide a full picture of how and why the adherence club intervention works (Figure 4). This is backed up by the respondents in the realist interviews. Most suggested that a combination of both theories could explain how the club intervention works. They used phrases like: ‘It is a combination of *both* your theories …’ [Nurse 1] or ‘I think both [*theories*]’ (Counsellor 2).

In the next step, we formulate a modified programme theory of the adherence club intervention based on the analysis of the Facility Y data set (see Box 2).

BOX 2 A modified programme theory of the adherence club intervention.

Grouping clinically stable patients on antiretroviral therapy **[Actors]** with available resources and buy-in from healthcare workers in a convenient space **[Context]** to receive a quick and uninterrupted supply of medication, health talks, counselling, immediate access to a clinician when required while guided by rules and regulations [**Intervention**], works because their self-efficacy improves and they become motivated and nudged **[Mechanisms]** to remain in care and adhere to medication [**Outcome**].

## Discussion

We aimed to confirm, refute or modify the initial programme theory of the adherence club intervention, which we drafted based on literature reviews and exploratory research. We examined the implementation of the adherence club intervention at Facility Y, chosen as a deviant case – poor performing based on 2014 routine data. Our hypothesis suggested two possible explanations of how and why the adherence club intervention improves retention in care and sustains adherence to medication: by motivating and empowering the patients towards adopting the desired behaviours or by nudging them into doing so.

These study findings showed that the two initial alternative theories complement each other to explain how and why the intervention works and in what context. Although some patients would become motivated and empowered, through improved self-efficacy to remain in care and adhere to their medication, others were *made* to remain in care by strictly enforcing rules and regulations of the adherence club programme. It is worth mentioning that different patients would respond better to different aspects of care embedded in the adherence club programme. Patients who already possess self-motivation would be empowered by the adherence club intervention as it enforces convenience to the patients. Other patients who may not be adequately self-motivated could respond better to being ‘told what to do’, which is the role that the rules and regulations of the adherence club programme plays, or fulfills. A combination of these two explanations provides a comprehensive understanding of how and why the adherence club intervention works. In another case study testing the initial programme theory, it was also confirmed that the combined programme theory explains how and why the adherence club enhances adherence to medication and promotes retention in care among stable patients on ART.^43^

Although the retention in care and the adherence rates of the patients at the facility seemed to have improved from 2014, as reflected in our current programme theory, it is worth exploring why the intervention failed to take off as intended. This could add value to understanding in what context or circumstances the intervention works or not. This follows the notion that programmes are open systems. By open system, realists argue that programmes cannot be fully isolated or kept constant and that they are affected by various conditions such as physical and technological shifts, personnel movements and learning, organisational imperative and so on.^16^ Such externalities always impact the delivery of a programme, and this entails that they are never quite implemented in the same way. In some instances, these externalities are introduced into the ‘system’ to engender a positive impact and are usually changes made to address the challenges that the intervention previously encountered.

The failure of the intervention to kick off was attributed to a myriad of factors, including lack of proper understanding of the adherence club programme, which led to poor buy-in from the healthcare providers and lack of required infrastructure. Buy-in, although considered an important mechanism at the level of implementing the intervention in the facility, nevertheless constitutes an important contextual factor regarding the day-to-day running as it affects the way the intervention is organised and delivered to the patients. Lack of buy-in was identified by the participants of the study as part of the reasons why the intervention was poorly implemented and executed at this facility. It was also mentioned that the operational staff failed to understand how the adherence club was going to work in their favour, and thus did not welcome the intervention until they were made to understand how it would decongest the healthcare facility. Because of the instructions received from sub-structure to roll out the adherence club programme in Facility Y, the operational staff working on the ART programme felt compelled to do something, although they did not share in the vision of the programme.

Our study unveiled that apart from the lack of a conducive space, impacting the buy-in of the healthcare providers, their understanding that the adherence club intervention would increase their workload also contributed to the diminishing buy-in. The notion of increased workload was compounded by perceived staff shortage. The perceived staff shortage also ties with the contextual factor of not having a programme champion to run the adherence club programme when it was first adopted by the facility in the first phased rollout.

Another important context element that was identified as a hindrance to the effective implementation and execution of the adherence club intervention at Facility Y was the lack of a conducive physical space where the meetings could be conducted. Our interviews revealed that when the intervention was rolled out at the facility, there was no physical space where the patients could meet to conduct the meetings. It took an intervention from the management to provide a makeshift building at the back of the facility for the intervention to be officially implemented. While exploring the context under which the adherence club is implemented in another facility, the authors found that lack of a conducive space for conducting club activities strongly influences the outcome of the intervention.^44^ This highlights the important role that context plays in activating the mechanisms that are provided by an intervention. In the absence of a conducive space, the buy-in of the healthcare providers became reduced, and they were not motivated to execute the intervention. This, in turn, affected the way the intervention was executed and the way it was received by the patients, thus impacting the retention in care and adherence outcomes. Dudhia and Kagee^45^ also uncovered that lack of resources for operating the club (delivery of care and support for club team) could demotivate the healthcare providers, thus impacting the quality of care delivered to patients.

The four important context conditions, lack of buy-in, lack of staff, the absence of a programme champion and lack of physical space for the club meetings, caused the mechanisms that are provided by the adherence club intervention and naturally occurring in the environment not to be triggered. Consequently, this led to sustained poor retention in care and suboptimal adherence to medication. While testing the initial programme theory in another context, we found that ‘integrating’ the adherence club programme with the management of patients with other non-communicable diseases (the presence of non-HIV-positive patients) presents a different prevailing context within which the adherence club intervention did not work.^44^ The context of integrated care was characterised by a lack of resources (adherence club meeting room), different execution models and poor adherence club programme coordination.^44^

When a nurse was identified and trained to champion the intervention, she exposed the other healthcare providers to the benefits of the intervention and headed the implementation. This engendered buy-in from the care workers. Following the buy-in, the pharmacists also reorganised their schedules to prepare medication packages for the club members. Once these elements were put in place, the context conditions of the adherence club changed, and the present conditions were favourable to incite the mechanisms provided by the adherence club intervention to cause the expected outcomes. These improved conditions and performance of the adherence club intervention are reflected in the high retention in care and adherence to medication rates, as demonstrated in the retrospective cohort analyses.

## Limitations, rigour and trustworthiness

Although viral load is commonly used as a proxy for ART adherence, it is not considered a perfect benchmark for evaluating how accurately an individual adheres to ART. This is especially true because for patients in the adherence club, their viral loads are only measured once a year. Thus, the viral load does not offer a real-time measure of adherence, which could be considered a limitation of the study.

Regarding the retention in care and adherence behaviours of patients in the adherence club, it would have been ideal to obtain the overall rates of the facility. This posed a challenge because the facility actively creates new clubs monthly. This would potentially affect the overall retention in care and adherence rates of patients in the adherence club programme. To this end, we decided to sample two adherence clubs that had reached their maximum capacity and to study the rate at which patients drop out of the club for various reasons – default, transferred out of the clinic, lost to follow-up or died.

To improve the rigour of the study, we adopted the mixed-method approach. The use of a multi-method approach to data collection was informed by its ability not only to improve the retroductive inferencing, but also to confirm and complement the information required to test the initial programme theory. In addition, we used a variety of participants to promote the triangulation of the information obtained from the participants as it makes it easy for the researchers to verify facts.

Frequent debriefing sessions were held among the authors. These sessions took place in all the phases of this study, including the data collection, analysis and synthesis phases.

## Conclusion

We conducted a theory-testing case study within the realist approach. We uncovered that patients on ART in adherence clubs will continue to adhere to their medication and remain in care because their self-efficacy is improved and they are motivated through the programme modalities and/or because they are being nudged through the club rules and regulations. Through the application of the realist evaluation approach, we modified the initial programme theory, which combines alternative theories to formulate a complementary theory. This is a step towards obtaining a refined programme theory of the adherence club intervention. With the adherence clubs currently being rolled out nationwide,^46^ understanding how, why, for whom and under what health systems context the adherence club programme works could inform its successful implementation to other contexts where it is required.

## References

[1] Human Sciences Research Council South African National HIV prevalence, incidence, behaviour and communication survey, 2017 [homepage on the Internet]. 2018 [cited 2019 Feb 22]. Available from: http://www.hsrc.ac.za/uploads/pageContent/9234/FINALPresentationfor17Julylaunch.pdf.

[2] Department of Health Implementation of the universal test and treat strategy for HIV positive patients and differentiated care for stable patients [homepage on the Internet]. Pretoria: Department of Health; 2016 [cited 2018 Oct 15]. Available from: http://www.sahivsoc.org/Files/22%208%2016%20Circular%20UTT%20%20%20Decongestion%20CCMT%20Directorate.pdf.

[3] Shisana O, Rhele T, Simbayi LC, et al The Human Sciences Research Council, 2012 2014.

[4] WHO Consolidated guidelines on the use of antiretroviral drugs for treating and preventing HIV infection: recommendations for a public health approach; 2016 [homepage on the Internet]. Switzerland: WHO; 2016 Available from: http://apps.who.int/iris/bitstream/10665/208825/1/9789241549684_eng.pdf?ua=1. 27466667

[5] Grimsrud A, Bygrave H, Doherty M, et al Reimagining HIV service delivery: The role of differentiated care from prevention to suppression. J Int AIDS Soc. 2016;19:21484 10.7448/IAS.19.1.21484 27914186 PMC5136137

[6] Wilkinson LS ART adherence clubs: A long-term retention strategy for clinically stable patients receiving antiretroviral therapy. S Afr J HIV Med. 2013;14(2):48–50. 10.7196/SAJHIVMED.924

[7] Bateman C MSF again paves the way with ART. S Afr Med J. 2013;103(2):71–73. 10.7196/SAMJ.6666 23374312

[8] Mukumbang FC, Marchal B, Van Belle S, Van Wyk B A realist approach to eliciting the initial programme theory of the antiretroviral treatment adherence club intervention in the Western Cape Province, South Africa. BMC Med Res Methodol. 2018;18:47 10.1186/s12874-018-0503-0 29801467 PMC5970495

[9] Luque-Fernandez MA, Van Cutsem G, Goemaere E, et al Effectiveness of patient adherence groups as a model of care for stable patients on antiretroviral therapy in Khayelitsha, Cape Town, South Africa. PLoS One. 2013;8(2):e56088 10.1371/journal.pone.0056088 23418518 PMC3571960

[10] Grimsrud A, Sharp J, Kalombo C, Bekker L-G, Myer L Implementation of community-based adherence clubs for stable antiretroviral therapy patients in Cape Town, South Africa. J Int AIDS Soc. 2015;18:19984 10.7448/IAS.18.1 26022654 PMC4444752

[11] Grimsrud A, Sharp J, Kalombo C, Bekker L-G, Myer L Outcomes of patients on antiretroviral therapy managed through community-based ‘Adherence Clubs’ in South Africa. J Acquir Immune Defic Syndr. 2016;71(1):e16–e23. 10.1097/QAI.0000000000000863 26473798

[12] Tsondai PR, Wilkinson LS, Grimsrud A, Mdlalo PT, Ullauri A, Boulle A High rates of retention and viral suppression in the scale-up of antiretroviral therapy adherence clubs in Cape Town, South Africa. J Int AIDS Soc. 2017;20(Suppl 4):51–57. 10.7448/IAS.20.5.21649 PMC5577696 28770595

[13] Bango F, Ashmore J, Wilkinson L, Van Cutsem G, Cleary S Adherence clubs for long-term provision of antiretroviral therapy: Cost-effectiveness and access analysis from Khayelitsha, South Africa. Trop Med Int Heal. 2016;21(9):1115–1123. 10.1111/tmi.12736 27300077

[14] Mukumbang FC, Van Belle S, Marchal B, Van Wyk B Realist evaluation of the antiretroviral treatment adherence club programme in selected primary healthcare facilities in the metropolitan area of Western Cape Province, South Africa: A study protocol. BMJ Open. 2016;6(4):e009977 10.1136/bmjopen-2015-009977 PMC4823437 27044575

[15] Kaboub F Realistic evaluation. Soc Sci J. 2004;41:153–154.

[16] Pawson R, Tilley N Realist evaluation [homepage on the Internet]. 2004 [cited 2018 Mar 23]. Available from: http://www.communitymatters.com.au/RE_chapter.pdf.

[17] Pawson R, Sridharan S Theory-driven evaluation of public health programmes Evidence-based public heal [homepage on the Internet]. Oxford University Press; 2009 [cited 2018 Mar 21], p. 43–62. Available from: http://www.oxfordscholarship.com/view/10.1093/acprof:oso/9780199563623.001.0001/acprof-9780199563623-chapter-04.

[18] Westhorp G, Stevens K, Rogers PJ Using realist action research for service redesign. Evaluation. 2016;22(3):361–379. 10.1177/1356389016656514

[19] Dalkin SM, Greenhalgh J, Jones D, Cunningham B, Lhussier M What’s in a mechanism? Development of a key concept in realist evaluation. Implement Sci. 2015;10:49 10.1186/s13012-015-0237-x 25885787 PMC4408605

[20] Mukumbang FC, Marchal B, Van Belle S, Van Wyk B Unearthing how, why, for whom and under what health system conditions the antiretroviral treatment adherence club intervention in South Africa works: A realist theory refining approach. BMC Health Serv Res. 2018;18(1):343 10.1186/s12913-018-3150-6 29743067 PMC5944119

[21] Marchal B, Westhorp G, Wong G, et al Realist RCTs of complex interventions – An oxymoron. Soc Sci Med. 2013;94(1):124–128. 10.1016/j.socscimed.2013.06.025 23850482

[22] Pawson R, Tilley N Realistic evaluation. 2nd ed. London: Sage; 1997.

[23] Mukumbang FC, Van Belle S, Marchal B, Van Wyk B Exploring ‘generative mechanisms’ of the antiretroviral adherence club intervention using the realist approach: A scoping review of research-based antiretroviral treatment adherence theories. BMC Public Health. 2017;17(1):385 10.1186/s12889-017-4322-8 28472938 PMC5418699

[24] Mukumbang FC, Van Belle S, Marchal B, Van Wyk B Towards developing an initial programme theory: Programme designers and managers assumptions on the antiretroviral treatment adherence club programme in primary health care facilities in the metropolitan area of western cape province, South Africa. PLoS One. 2016;11(8):e0161790 10.1371/journal.pone.0161790 27560352 PMC4999218

[25] Mukumbang FC, Van Belle S, Marchal B, Van Wyk B An exploration of group-based HIV/AIDS treatment and care models in Sub-Saharan Africa using a realist evaluation (intervention-context-actor-mechanism-outcome) heuristic tool: A systematic review. Implement Sci. 2017;12:107 10.1186/s13012-017-0638-0 28841894 PMC5574210

[26] Wong G, Westhorp G, Manzano A, et al RAMESES II reporting standards for realist evaluations. BMC Med. 2016;14(1):96 10.1186/s12916-016-0643-1 27342217 PMC4920991

[27] Yin R Case study research: Design and methods. Los Angeles, CA: Sage; 2014.

[28] Creswell JW, Plano Clark VL Designing and conducting mixed methods research. London: Sage; 2011.

[29] Bryman A Integrating quantitative and qualitative research: How is it done? Qual Res. 2006;6(1):97–113. 10.1177/1468794106058877

[30] Punsalan T, Uriarte G Statistics: A simplified approach. In: Ochave JA, Seville CG, editors 1st ed. Manila: Rex Book Store; 1987;1–207.

[31] Mills AJ, Eurepos G, Wiebe E Encyclopedia of case study research. London: Sage; 2010.

[32] Pawson R Theorizing the interview. Br J Sociol. 1996;47:295–314.

[33] Manzano A The craft of interviewing in realist evaluation. Evaluation. 2016;22(3):342–360. 10.1177/1356389016638615

[34] Bland JM, Altman DG Survival probabilities (the Kaplan–Meier method). BMJ. 1998;317(7172):1572. 9836663 10.1136/bmj.317.7172.1572 PMC1114388

[35] Campbell JL, Quincy C, Osserman J, Pedersen OK Coding in-depth semistructured interviews: Problems of unitization and intercoder reliability and agreement. Sociol Methods Res. 2013;42(2):294–320. 10.1177/0049124113500475

[36] Lee TY, Lok DPP Bonding as a positive youth development construct: A conceptual review. Sci World J. 2012;2012:11 10.1100/2012/481471 PMC3353469 22623898

[37] Bandura A Social cognitive theory: An agentic perspective. Annu Rev Psychol. 2001;6:1–60. 10.1146/annurev.psych.52.1.1 11148297

[38] Fox MP, Bor J, Brennan AT, et al Estimating retention in HIV care accounting for patient transfers: A national laboratory cohort study in South Africa. PLoS Med. 2018;15:30–43. 10.1371/journal.pmed.1002589 PMC5995345 29889844

[39] Wynn D, Williams CK Principles for conducting critical realist case study research in information systems. MIS Q. 2012;36(3):787–810.

[40] Eastwood JG, Jalaludin BB, Kemp LA Realist explanatory theory building method for social epidemiology: A protocol for a mixed method multilevel study of neighbourhood context and postnatal depression. Springerplus. 2014;3:12 10.1186/2193-1801-3-12 24422187 PMC3888492

[41] Ferraro PJ Counterfactual thinking and impact evaluation in environmental policy. Environmental program and policy evaluation. New Dir Eval. 2009;122(112):75–84. 10.1002/ev.297

[42] Byng R, Norman I, Redfern S Using realistic evaluation to evaluate a practice-level intervention to improve primary health care for patients with long-term mental illness. Evaluation. 2005;11(1):69–93. 10.1177/1356389005053198

[43] Mukumbang FC, Van Wyk B, Van Belle S, Marchal B Unravelling how and why the Antiretroviral Adherence Club Intervention works (or not) in a public health facility: A realist explanatory theory-building case study. PLoS One. 2019;14(1):e0210565 10.1371/journal.pone.0210565 30650129 PMC6334969

[44] Mukumbang FC, Marchal B, Van Belle S, Van Wyk B ‘Patients are not following the [Adherence] club rules anymore’: A realist case study of the antiretroviral treatment adherence club, South Africa. Qual Health Res. 2018;18(12):1839–1857. 10.1177/1049732318784883 PMC6154254 30033857

[45] Dudhia R, Kagee A Experiences of participating in an antiretroviral treatment adherence club. Psychol Heal Med. 2015;20(4):488–494. 10.1080/13548506.2014.953962 PMC4550101 25168720

[46] Mukumbang FC, Zaida O, Van Wyk B What do the implementation outcome variables tell us about the scaling-up of the antiretroviral treatment adherence clubs in South Africa? A document review. Health Res Policy Syst. 2019;17:28 10.1186/s12961-019-0428-z 30871565 PMC6419395

